# Ease of use of the ELLIPTA dry powder inhaler: data from three randomised controlled trials in patients with asthma

**DOI:** 10.1038/npjpcrm.2014.19

**Published:** 2014-06-26

**Authors:** Henrik Svedsater, Loretta Jacques, Caroline Goldfrad, Eugene R Bleecker

**Affiliations:** 1 Value Evidence and Outcomes, GlaxoSmithKline, Stockley Park, Uxbridge, UK; 2 Respiratory Medicines Development Centre, GlaxoSmithKline, Uxbridge, UK; 3 Quantitative Sciences Division, GlaxoSmithKline, Uxbridge, UK; 4 Center for Genomics and Personalized Medicine, Wake Forest School of Medicine, Winston-Salem, NC, USA

Maintenance therapies for asthma are typically delivered via handheld inhalers. Poor adherence to inhaled medications and incorrect inhaler technique are known to adversely affect outcomes in asthma, contributing to the continuing failure for many patients to achieve control despite the availability of effective therapies.^1^

The ELLIPTA dry powder inhaler (DPI) is a handheld inhaler with single-step activation, featuring a cover that is opened by the patient to uncover the mouthpiece and activate a dose^2^ (ELLIPTA is a trademark of the GlaxoSmithKline group of companies). The actuated dose is subsequently inhaled from the mouthpiece.^2^ The ELLIPTA DPI is used to deliver fluticasone furoate (FF), a new inhaled corticosteroid licensed in Europe in combination with vilanterol, a new long-acting β_2_-agonist, for asthma and chronic obstructive pulmonary disease and in development as a monotherapy for asthma. The aim of this analysis was to investigate patient perception of the ease of use, and investigator-reported competence in use, of the ELLIPTA DPI.

We describe a sub-analysis of the ease of use and inhaler competence data in patients with asthma from three randomised, multicentre clinical trials of FF/vilanterol combination therapy (HZA106827 (100/25 μg) and/or FF monotherapy (FFA114496 (100, 200 μg); FFA115283 (50 μg); HZA106827 (100 μg)), in which the ELLIPTA DPI was used to deliver study medication (including placebo where applicable). Preliminary results have been published in abstract form.^2^ The primary clinical trial data are reported separately.^3–5^

Patients completed a questionnaire at week 4 of each trial, rating the ease of use of the inhaler and how easy it was to tell how many doses of medication were left in the inhaler. For both questions, patients selected their response from the following ordinal scale: very easy, easy, neutral, difficult, and very difficult. Investigators assessed, by observation, patients’ competence in using the ELLIPTA DPI following one demonstration of correct usage at randomisation, at week 2 and at week 4. Data were analysed and interpreted descriptively; no statistical inference was planned.

A total of 1,050 asthma patients (Supplementary Appendix) participated in the trials. Of these, 94% completed the questionnaire. Patient-reported ease of use and investigator-reported inhaler use assessment findings for each of the three clinical trials, together with pooled results, are presented in Table 1. The findings of both assessments were similar across the three trials (Supplementary Appendix).

Overall, 65% of questionnaire respondents reported that the inhaler was very easy to use, and 94% reported that it was easy or very easy to use. Only 1% of patients reported that the inhaler was difficult or very difficult to use. Similarly, 74% reported that they found it very easy to tell how many doses of medication were left in the inhaler using the in-built numerical dose counter, and 96% found it easy or very easy. Less than 1% of patients found it difficult or very difficult to tell how many doses were left in the inhaler.

At randomisation, investigators reported that 95% of patients used the inhaler correctly after the initial demonstration of correct usage at randomisation (week 0), and did not require additional instruction. A further 4% of patients were able to use the inhaler correctly at randomisation after one additional instruction. The most common error made at randomisation (before any additional instruction) was to open the cover incorrectly (20 (1.9%) of all patients), followed by inhaling the dose incorrectly (15 (1.4%)), unspecified reason (12 (1.1%)), and closing the cover incorrectly (3 (0.3%)). At week 2 and week 4, >99% of patients used the inhaler correctly; four (0.4%) patients made errors at week 2 and week 4, respectively (Supplementary Appendix).

In all three studies, the majority of participants found the inhaler to be easy to use, and were observed to use the inhaler correctly following a single demonstration. The design of the inhaler and appropriateness of the delivery mechanism to the patient may boost patient satisfaction with the medication regimen and competence in device use.^6^ Patient preference data obtained from a separate interview-based study^7^ are consistent with our findings, suggesting that patients with asthma and chronic obstructive pulmonary disease generally perceive the ELLIPTA DPI positively and find it easy to use.

A similar questionnaire has previously been used to assess comparative ease of use in asthma patients participating in randomised controlled trials, whose responses indicated that the DISKUS DPI is easier to use than DiskHaler.^8,9^ Similarly, our single-device study was conducted against the background of randomised, controlled clinical trials in which all patients were given clear instruction in correct use of the inhaler at randomisation. Such thorough instruction is unlikely to be replicated in real-world clinical practice;^10^ this could therefore be considered a limitation in interpretation of this study.

The perceived and observed ease of use findings reported in this analysis suggest that the ELLIPTA DPI may have the potential to reduce inhaler-related handling errors and improve adherence; however, further studies are required to specifically assess these possibilities.

## Figures and Tables

**Table 1:** Summary of findings of ELLIPTA dry powder inhaler ease of use questionnaire and investigator assessment of inhaler technique for each study and when pooled together

	*HZA106827* N*=609*	*FFA114496* N*=219*	*FFA115283* N*=222*	*Total* N*=1,050*
*Ease of use questionnaire*	*n*=570	*n*=213	*n*=206	*N*=989
				
*How did you rate the ease of use of the inhaler?, n (%)*				
Very easy	362 (64)	146 (69)	132 (64)	640 (65)
Easy	157 (28)	64 (30)	68 (33)	289 (29)
Neutral	43 (8)	3 (1)	4 (2)	50 (5)
Difficult	7 (1)	0	2 (<1)	9 (1)
Very difficult	1 (<1)	0	0	1 (<1)
				
*How easily are you able to tell how many doses of medication are left in the inhaler?, n (%)*				
Very easy	419 (74)	169 (79)	144 (70)	732 (74)
Easy	126 (22)	42 (20)	51 (25)	219 (22)
Neutral	22 (4)	2 (<1)	8 (4)	32 (3)
Difficult	3 (<1)	0	1 (<1)	4 (<1)
Very difficult	0	0	2 (<1)	2 (<1)
				
*Inhaler use assessment*				
Randomisation,^a^ *n* (%)				
*n*	609	218	222	1,049
Patient used the inhaler correctly	578 (95)	206 (94)	216 (97)	1,000 (95)
1 Additional instruction required	22 (4)	11 (5)	5 (2)	38 (4)
2 Additional instructions required	8 (1)	1 (<1)	1 (<1)	10 (1)
3 Additional instructions required	1 (<1)	0	0	1 (<1)
>3 Additional instructions required	0	0	0	0
Week 2, *n* (%)				
*n*	593	215	216	1,024
Patient used the inhaler correctly	593 (100)	211 (98)	216 (100)	1,020 (>99)
1 Additional instruction required	0	3 (1)	0	3 (<1)
2 Additional instructions required	0	1 (<1)	0	1 (<1)
3 Additional instructions required	0	0	0	0
>3 Additional instructions required	0	0	0	0
Week 4, *n* (%)				
*n*	569	213	206	988
Patient used the inhaler correctly	569 (100)	210 (99)	205 (>99)	984 (>99)
1 Additional instruction required	0	3 (1)	1 (<1)	4 (<1)
2 Additional instructions required	0	0	0	0
3 Additional instructions required	0	0	0	0
>3 Additional instructions required	0	0	0	0

a Week 0; after one demonstration of correct usage.

## Notes

HS, LJ and CG are employees of and hold stock in GlaxoSmithKline. ERB has served as a consultant for AstraZeneca, Boehringer Ingelheim, Genentech, GlaxoSmithKline, Johnson and Johnson, Merck, Regeneron, Sanofi-Aventis and Forest, and has performed clinical trials for AstraZeneca, Boehringer Ingelheim, Cephalon, Forest, Genentech, GlaxoSmithKline, KalaBios, MedImmune, Novartis and Sanofi-Aventis, which have been administered by his employer Wake Forest University School of Medicine.
