# Supplementary material for: Ease of use of the ELLIPTA dry powder inhaler: data from three randomised controlled trials in patients with asthma
Source: NPJ Prim Care Respir Med. 2014 Jun 26;24:14019–. doi: 10.1038/npjpcrm.2014.19 (PMC4373320; doi:10.1038/npjpcrm.2014.19)
Supplement: Supplementary Appendix [file npjpcrm201419-s1.doc]

**Appendix – Svedsater *et al***

e-table 1 – Patient baseline demographics/characteristics

|  | **HZA106827** | **FFA114496** | **FFA115283** |
| --- | --- | --- | --- |
| **ITT population, N**  Receiving FF/VI, n  Receiving FF, n  Receiving placebo, n | **609**  201^a^  205^b^  203 | **219**  —  219^c^  — | **222**  —  111^d^  111 |
| **Age, years** | 39.7 (16.56) | 46.4 (15.42) | 35.2 (15.11) |
| **Sex, female** | 353 (58) | 148 (68) | 133 (60) |
| **Duration of asthma, years** | 12.11 (11.435) | 20.49 (15.545) | 16.59 (11.972) |
| **Study site location, n (%)**  Argentina  Chile  France  Germany  Japan  Mexico  Poland  Peru  Romania  Russian Federation  Ukraine  United States | —  —  —  67 (11)  50 (8)  —  124 (20)  —  89 (15)  —  83 (14)  196 (32) | 83 (38)  38 (17)  1 (<1)  —  —  23 (11)  —  —  —  38 (17)  —  36 (16) | —  —  —  —  —  63 (28)  —  84 (38)  —  38 (17)  —  37 (17) |
| **FEV_1_, % reversibility at screening** | 28.71 (18.254) | 32.27 (18.549) | 24.54 (10.119) |
| **Pre-bronchodilator FEV_1_ at baseline, L** | 2.323 (0.6275) | 2.061 (0.6599) | 2.690 (0.8223) |
| **FEV_1_, % predicted at baseline** | 70.43 (11.014) | 68.11 (13.619) | 81.58 (13.218) |

All data are mean (SD) unless otherwise stated

^a^201 patients received FF/VI 100/25µg once daily

^b^205 patients received FF 100µg once daily

^c^108 patients received FF 100µg once daily and 111 patients received FF 200µg once daily

^d^111 patients received FF 50µg once daily

FEV_1_: forced expiratory volume in one second; FF: fluticasone furoate; ITT: intent-to-treat; VI: vilanterol

e-table 2 – Full findings from inhaler use assessment and patient-reported ease of use questionnaire – HZA106827

| Randomisation | Placebo  (N=203) | FF 100 OD  (N=205) | FF/VI 100/25 OD  (N=201) | Total  (N=609) |
| --- | --- | --- | --- | --- |
| **Did the patient use the Inhaler correctly?** |  |  |  |  |
| n | 203 | 205 | 201 | 609 |
| Yes | 194 (96%) | 196 (96%) | 188 (94%) | 578 (95%) |
| No | 9 (4%) | 9 (4%) | 13 (6%) | 31 (5%) |
| **If no, what did the patient do incorrectly?** |  |  |  |  |
| n | 9 | 9 | 13 | 31 |
| Open the device | 8 (89%) | 4 (44%) | 6 (46%) | 18 (58%) |
| Inhale the dose | 1 (11%) | 5 (56%) | 6 (46%) | 12 (39%) |
| Close the device | 1 (11%) | 0 | 1 (8%) | 2 (6%) |
| **Number of times the patient required additional instruction** |  |  |  |  |
| n | 9 | 9 | 13 | 31 |
| 1 | 9 (100%) | 5 (56%) | 8 (62%) | 22 (71%) |
| 2 | 0 | 4 (44%) | 4 (31%) | 8 (26%) |
| 3 | 0 | 0 | 1 (8%) | 1 (3%) |
| >3 | 0 | 0 | 0 | 0 |

| Week 2 | Placebo  (N=203) | FF 100 OD  (N=205) | FF/VI 100/25 OD  (N=201) | Total  (N=609) |
| --- | --- | --- | --- | --- |
| **Did the patient use the Inhaler correctly?** |  |  |  |  |
| n | 190 | 203 | 200 | 593 |
| Yes | 190 (100%) | 203 (100%) | 200 (100%) | 593 (100%) |
| No | 0 | 0 | 0 | 0 |
| **If no, what did the patient do incorrectly?** |  |  |  |  |
| n | 0 | 0 | 0 | 0 |
| Open the device | 0 | 0 | 0 | 0 |
| Inhale the dose | 0 | 0 | 0 | 0 |
| Close the device | 0 | 0 | 0 | 0 |
| **Number of times the patient required additional instruction** |  |  |  |  |
| n | 0 | 0 | 0 | 0 |
| 1 | 0 | 0 | 0 | 0 |
| 2 | 0 | 0 | 0 | 0 |
| 3 | 0 | 0 | 0 | 0 |
| >3 | 0 | 0 | 0 | 0 |

| Week 4 | Placebo  (N=203) | FF 100 OD  (N=205) | FF/VI 100/25 OD  (N=201) | Total  (N=609) |
| --- | --- | --- | --- | --- |
| **Did the patient use the Inhaler correctly?** |  |  |  |  |
| n | 175 | 199 | 195 | 569 |
| Yes | 175 (100%) | 199 (100%) | 195 (100%) | 569 (100%) |
| No | 0 | 0 | 0 | 0 |
| **If no, what did the patient do incorrectly?** |  |  |  |  |
| n | 0 | 0 | 0 | 0 |
| Open the device | 0 | 0 | 0 | 0 |
| Inhale the dose | 0 | 0 | 0 | 0 |
| Close the device | 0 | 0 | 0 | 0 |
| **Number of times the patient required additional instruction** |  |  |  |  |
| n | 0 | 0 | 0 | 0 |
| 1 | 0 | 0 | 0 | 0 |
| 2 | 0 | 0 | 0 | 0 |
| 3 | 0 | 0 | 0 | 0 |
| >3 | 0 | 0 | 0 | 0 |

| Week 4 | Placebo  (N=203) | FF 100 OD  (N=205) | FF/VI 100/25 OD  (N=201) | Total  (N=609) |
| --- | --- | --- | --- | --- |
| **How do you rate the ease of use of the inhaler?** |  |  |  |  |
| n | 176 | 199 | 195 | 570 |
| Very Easy | 107 (61%) | 126 (63%) | 129 (66%) | 362 (64%) |
| Easy | 53  (30%) | 50  (25%) | 54  (28%) | 157 (28%) |
| Neutral | 13 (7%) | 19 (10%) | 11 (6%) | 43 (8%) |
| Difficult | 2 (1%) | 4 (2%) | 1 (<1%) | 7 (1%) |
| Very Difficult | 1 (<1%) | 0 | 0 | 1 (<1%) |
| **How easily are you able to tell how many doses of medication are left in the inhaler?** |  |  |  |  |
| n | 176 | 199 | 195 | 570 |
| Very Easy | 126 (72%) | 146 (73%) | 147 (75%) | 419 (74%) |
| Easy | 41  (23%) | 46  (23%) | 39  (20%) | 126 (22%) |
| Neutral | 8 (5%) | 6 (3%) | 8 (4%) | 22 (4%) |
| Difficult | 1 (<1%) | 1 (<1%) | 1 (<1%) | 3 (<1%) |
| Very Difficult | 0 | 0 | 0 | 0 |

FF = fluticasone furoate; OD = once-daily; VI = vilanterol

e-table 3 - Full findings from inhaler use assessment and patient-reported ease of use questionnaire – FFA115283

| Randomisation | Placebo  (N=111) | FF 50 OD  (N=111) | Total  (N=222) |
| --- | --- | --- | --- |
| **Did the patient use the Inhaler correctly?** |  |  |  |
| n | 111 | 111 | 222 |
| Yes | 107 (96%) | 109 (98%) | 216 (97%) |
| No | 4 (4%) | 2 (2%) | 6 (3%) |
| **If no, what did the patient do incorrectly?** |  |  |  |
| n | 1 | 1 | 2 |
| Open the device | 1 (100%) | 1 (100%) | 2 (100%) |
| Inhale the dose | 0 | 0 | 0 |
| Close the device | 0 | 0 | 0 |
| **Number of times the patient required additional instruction** |  |  |  |
| n | 4 | 2 | 6 |
| 1 | 4 (100%) | 1 (50%) | 5 (83%) |
| 2 | 0 | 1 (50%) | 1 (17%) |
| 3 | 0 | 0 | 0 |
| >3 | 0 | 0 | 0 |

| Week 2 | Placebo  (N=111) | FF 50 OD  (N=111) | Total  (N=222) |
| --- | --- | --- | --- |
| **Did the patient use the Inhaler correctly?** |  |  |  |
| n | 108 | 108 | 216 |
| Yes | 108 (100%) | 108 (100%) | 216 (100%) |
| No | 0 |  | 0 |
| **If no, what did the patient do incorrectly?** |  |  |  |
| n | 0 | 0 | 0 |
| Open the device | 0 | 0 | 0 |
| Inhale the dose | 0 | 0 | 0 |
| Close the device | 0 | 0 | 0 |
| **Number of times the patient required additional instruction** |  |  |  |
| n | 0 | 0 | 0 |
| 1 | 0 | 0 | 0 |
| 2 | 0 | 0 | 0 |
| 3 | 0 | 0 | 0 |
| >3 | 0 | 0 | 0 |

| Week 4 | Placebo  (N=111) | FF 50 OD  (N=111) | Total  (N=222) |
| --- | --- | --- | --- |
| **Did the patient use the Inhaler correctly?** |  |  |  |
| n | 99 | 107 | 206 |
| Yes | 99 (100%) | 106 (>99%) | 205 (>99%) |
| No | 0 | 1 (<1%) | 1 (<1%) |
| **If no, what did the patient do incorrectly?** |  |  |  |
| n | 0 | 1 | 1 |
| Open the device | 0 | 1 (100%) | 1 (100%) |
| Inhale the dose | 0 | 0 | 0 |
| Close the device | 0 | 0 | 0 |
| **Number of times the patient required additional instruction** |  |  |  |
| n | 0 | 1 | 1 |
| 1 | 0 | 1 (100%) | 1 (100%) |
| 2 | 0 | 0 | 0 |
| 3 | 0 | 0 | 0 |
| >3 | 0 | 0 | 0 |

| Week 4 | Placebo  (N=111) | FF 50 OD  (N=111) | Total  (N=222) |
| --- | --- | --- | --- |
| **How do you rate the ease of use of the inhaler?** |  |  |  |
| n | 99 | 107 | 206 |
| Very Easy | 59 (60%) | 73 (68%) | 132 (64%) |
| Easy | 38 (38%) | 30 (28%) | 66 (33%) |
| Neutral | 2 (2%) | 2 (2%) | 4 (2%) |
| Difficult | 0 | 2 (2%) | 2 (<1%) |
| Very Difficult | 0 | 0 | 0 |
| **How easily are you able to tell how many doses of medication are left in the inhaler?** |  |  |  |
| n | 99 | 107 | 206 |
| Very Easy | 65 (66%) | 79 (74%) | 144 (70%) |
| Easy | 25 (25%) | 26 (24%) | 51 (25%) |
| Neutral | 7 (7%) | 1 (<1%) | 8 (4%) |
| Difficult | 0 | 1 (<1%) | 1 (<1%) |
| Very Difficult | 2 (2%) | 0 | 2 (<1%) |

FF = fluticasone furoate; OD = once-daily; VI = vilanterol

**e-table 4 - Full findings from inhaler use assessment and patient-reported ease of use questionnaire – FFA114496**

| Randomisation | FF 100 OD  (N=108) | FF 50 OD  (N=111) | Total  (N=219) |
| --- | --- | --- | --- |
| **Did the patient use the Inhaler correctly?** |  |  |  |
| n | 107 | 111 | 218 |
| Yes | 102 (95%) | 104 (94%) | 206 (94%) |
| No | 5 (5%) | 7 (6%) | 12 (6%) |
| **If no, what did the patient do incorrectly?** |  |  |  |
| n | 1 | 3 | 4 |
| Open the device | 0 | 0 | 0 |
| Inhale the dose | 1 (100%) | 2 (67%) | 3 (75%) |
| Close the device | 0 | 1 (33%) | 1 (25%) |
| **Number of times the patient required additional instruction** |  |  |  |
| n | 5 | 7 | 12 |
| 1 | 5 (100%) | 6 (86%) | 11 (92%) |
| 2 | 0 | 1 (14%) | 1 (8%) |
| 3 | 0 | 0 | 0 |
| >3 | 0 | 0 | 0 |

| Week 2 | FF 100 OD  (N=108) | FF 50 OD  (N=111) | Total  (N=219) |
| --- | --- | --- | --- |
| **Did the patient use the Inhaler correctly?** |  |  |  |
| n | 106 | 109 | 215 |
| Yes | 102 (96%) | 109 (100%) | 211 (98%) |
| No | 4 (4%) | 0 | 4 (2%) |
| **If no, what did the patient do incorrectly?** |  |  |  |
| n | 3 | 0 | 3 |
| Open the device | 2 (67%) | 0 | 2 (67%) |
| Inhale the dose | 0 | 0 | 0 |
| Close the device | 1 (33%) | 0 | 1 (33%) |
| **Number of times the patient required additional instruction** |  |  |  |
| n | 4 | 0 | 4 |
| 1 | 3 (75%) | 0 | 3 (75%) |
| 2 | 1 (25%) | 0 | 1 (25%) |
| 3 | 0 | 0 | 0 |
| >3 | 0 | 0 | 0 |

| Week 4 | FF 100 OD  (N=108) | FF 50 OD  (N=111) | Total  (N=219) |
| --- | --- | --- | --- |
| **Did the patient use the Inhaler correctly?** |  |  |  |
| n | 105 | 108 | 213 |
| Yes | 103 (98%) | 107 (>99%) | 210 (99%) |
| No | 2 (2%) | 1 (<1%) | 3 (1%) |
| **If no, what did the patient do incorrectly?** |  |  |  |
| n | 2 | 1 | 3 |
| Open the device | 2 (100%) | 1 (100%) | 3 (100%) |
| Inhale the dose | 0 |  | 0 |
| Close the device | 0 |  | 0 |
| **Number of times the patient required additional instruction** |  |  |  |
| n | 2 | 1 | 3 |
| 1 | 2 (100%) | 1 (100%) | 3 (100%) |
| 2 | 0 | 0 | 0 |
| 3 | 0 | 0 | 0 |
| >3 | 0 | 0 | 0 |

| Week 4 | FF 100 OD  (N=108) | FF 50 OD  (N=111) | Total  (N=219) |
| --- | --- | --- | --- |
| **How do you rate the ease of use of the inhaler?** |  |  |  |
| n | 105 | 108 | 213 |
| Very Easy | 75 (71%) | 71 (66%) | 146 (69%) |
| Easy | 28 (27%) | 36 (33%) | 64 (30%) |
| Neutral | 2 (2%) | 1 (<1%) | 3 (1%) |
| Difficult | 0 | 0 | 0 |
| Very Difficult | 0 | 0 | 0 |
| **How easily are you able to tell how many doses of medication are left in the inhaler?** |  |  |  |
| n | 105 | 108 | 213 |
| Very Easy | 88 (84%) | 81 (75%) | 169 (79%) |
| Easy | 16 (15%) | 26 (24%) | 42 (20%) |
| Neutral | 1 (<1%) | 1 (<1%) | 2 (<1%) |
| Difficult | 0 | 0 | 0 |
| Very Difficult | 0 | 0 | 0 |

FF = fluticasone furoate; OD = once-daily; VI = vilanterol
